# Menstrual Leucorrhœa Contra Indications for Vaginal Injections

**Published:** 1884-06-20

**Authors:** Stoyell C. Parsons

**Affiliations:** Physician to the Northeastern Dispensary


### Southern Medical Record:

EDITORS:

THOMAS S. POWELL, M.D. R. C. WORD, M.D.

*R. C. WORD, M.D., Managing Editor.*

☞All Communications and Letters on Business connected with the RECORD must be addressed to the Managing Editor.

VOL. XIV. ATLANTA, GA., JUNE 20, 1884. No. 6.

### ORIGINAL AND SELECTED ARTICLES.

### MENSTRUAL LEUCORRHŒA CONTRA INDICATIONS FOR VAGINAL INJECTIONS.

BY STOYELL C. PARSONS, M. S., M. D.,
Physician to the Northeastern Dispensary.

The frequency with which you meet unmarried females afflicted with menstrual leucorrhœa under treatment identically the same as though originating from other causes, has been a constant matter of surprise to me, as they necessarily require one altogether different in consequence of the surroundings to which they are subjected.

There should be as much attention given in every instance to the proper analysis of the symptoms as is exercised by the lawyer for each client, and when so considered, the physician will concur with the attorney that the case is entirely distinct and separate, regardless of the close similarity of all the signs that may be presented by the disease.

Menstrual leucorrhœa is defined as a discharge that to a greater or less degree, takes the place of the menses.

Such being the complaint, the plan of treatment that seems to be the most practical, is to turn our attention to the cause, and endeavor to establish the menses in quantity both sufficient and regular, and not to treat the leucorrhœa as a simple local affair by ablutions and astringents. When treated by astringent injections we find that it develops into a long and chronic complaint, and instead of relieving, we aggravate her symptoms in consequence of the undue excitability caused by the friction of the liquid and the pipe of the syringe.

I will quote one case as an instance :

Miss Mary M———, age 23, complaining of general malaise, backache, pain in left side and abdomen, subsequently located over the ovaries, and a slight leucorrhœa, applies to her physician and is ordered to use as an injection daily in the morning a solution of alum, with the strength of a drachm to the pint of tepid water.

Not recovering she consults another physician; is examined and her case is diagnosed retroversion with leucorrhœa, with the advice to continue the same treatment, and in addition use the injection in the evening before retiring.

The treatment was continued for a period of three months and then came under my observation, with the same symptoms of her original complaint considerably aggravated, and in addition suffering from melancholia, occipital headache, lassitude, and lascivious dreams accompanied with an orgasm most every night, while she was also liable to have an orgasm in the presence of men.

She states that the using of the syringe excites her venereal passions, and, that previous to the use of the same, she suffered none whatever from any of the latter symptoms which have been developed in a period of about a year.

There are quite a large number of married, and a larger percentage of widows which object to vaginal injections on account of its being "so disagreeable" as they term it, and when the cause is traced out, it results in finding that it is in consequence of arousing a passion that they do not wish or can not gratify.

In every instance the use of the syringe has been the means of continuing and aggravating the leucorrhœa, and by the constant irritation developed the more serious symptoms as heretofore described.

